# Parasitic Manipulation of Host Behaviour: Baculovirus SeMNPV EGT Facilitates Tree-Top Disease in *Spodoptera exigua* Larvae by Extending the Time to Death

**DOI:** 10.3390/insects6030716

**Published:** 2015-07-31

**Authors:** Yue Han, Stineke van Houte, Gerben F. Drees, Monique M. van Oers, Vera I. D. Ros

**Affiliations:** 1 Laboratory of Virology, Wageningen University, Droevendaalsesteeg 1, 6708 PB Wageningen, the Netherlands; E-Mails: yue.han@wur.nl (Y.H.); vanhoute.stineke@gmail.com (S.V.H.); gerben0811@gmail.com (G.F.D.); vera.ros@wur.nl (V.I.D.R.); 2 Centre for Ecology and Conservation, Biosciences, University of Exeter, Penryn, Cornwall TR10 9FE, UK

**Keywords:** behavioural manipulation, baculovirus, tree-top disease, SeMNPV, *egt* gene, *Spodoptera exigua*

## Abstract

Many parasites enhance their dispersal and transmission by manipulating host behaviour. One intriguing example concerns baculoviruses that induce hyperactivity and tree-top disease (*i.e.*, climbing to elevated positions prior to death) in their caterpillar hosts. Little is known about the underlying mechanisms of such parasite-induced behavioural changes. Here, we studied the role of the ecdysteroid UDP-glucosyltransferase (*egt*) gene of *Spodoptera exigua* multiple nucleopolyhedrovirus (SeMNPV) in tree-top disease in *S. exigua* larvae. Larvae infected with a mutant virus lacking the *egt* gene exhibited a shorter time to death and died before the induction of tree-top disease. Moreover, deletion of either the open reading frame or the ATG start codon of the *egt* gene prevented tree-top disease, indicating that the EGT protein is involved in this process. We hypothesize that SeMNPV EGT facilitates tree-top disease in *S. exigua* larvae by prolonging the larval time to death. Additionally, we discuss the role of *egt* in baculovirus-induced tree-top disease.

## 1. Introduction

A wide range of parasites are able to modify the behaviour of their hosts upon infection. Intriguing examples include the suicidal behaviour of the cricket *Nemobius sylvestris* when parasitized by the hairworm *Paragordius tricuspidatus* [1] and the long-time hypokinetic stage of the cockroach *Periplaneta americana* when stung by the parasitoid jewel wasp *Ampulex compressa* [2]. Many parasite-induced behavioural changes are thought to enhance parasite reproduction, transmission and/or survival [3,4,5]. Baculoviruses are large, double-stranded DNA viruses that induce behavioural changes in caterpillars. Infected host caterpillars show hyperactivity, which may spread viral progeny over larger areas [6,7,8]. Additionally, infected larvae present atypical climbing behaviour prior to death, leading to their migration to the top of plants. This pre-death climbing behaviour is described as “Wipfelkrankheit” or “tree-top disease” [8,9,10,11]. The resulting increased visibility of infected insects to predators may enhance long-distance virus dispersal [12] and the final liquefaction of larval cadavers at elevated positions promotes virus dissemination over the foliage [8].

Unravelling the underlying molecular mechanisms that drive baculovirus-induced host behavioural changes is the main focus of several recent studies. A significant study by Kamita *et al.* [7] showed that the protein tyrosine phosphatase *(ptp)* gene from the baculovirus *Bombyx mori* nucleopolyhedrovirus (BmNPV) is involved in inducing hyperactivity in *B. mori* silkworms. Similarly, the *Autographa californica* multiple nucleopolyhedrovirus (AcMNPV) *ptp* gene is required for induction of hyperactivity in *Spodoptera exigua* caterpillars [6]. Because both BmNPV and AcMNPV belong to the same taxon of baculoviruses (the Group I NPVs in the genus *Alphabaculovirus*), it is hypothesized that *ptp*-induced hyperactivity is a conserved strategy among Group I NPVs to enhance virus transmission [6]. Another study by Hoover *et al.* [9] showed that the ecdysteroid uridine 5'-diphosphate (UDP)-glucosyltransferase (*egt*) gene from *Lymantria dispar* MNPV (LdMNPV) is required for triggering tree-top disease in gypsy moth larvae. However, it seems that the role of the *egt* gene in inducing tree-top disease varies between virus-host combinations. Unlike the *egt* gene from LdMNPV, the *egt* gene from AcMNPV is not needed for inducing tree-top disease in *S. exigua* and *Trichoplusia ni* larvae: when the *egt* gene was deleted from the AcMNPV genome, infected larvae still died at elevated positions [10]. In addition, the *egt* gene seems not to be involved in the induction of hyperactivity: hyperactivity is still triggered in *egt-*negative BmNPV-infected *B. mori* larvae [13]. On the other hand, the AcMNPV *ptp* gene is not involved in inducing tree-top disease in *S. exigua* larvae [14]. Therefore, AcMNPV-induced hyperactivity and tree-top disease are mediated by different mechanisms. Whether the baculovirus *ptp2* gene [6], which is distantly related to the AcMNPV *ptp* gene, has a role in behavioural manipulation is not known.

During baculovirus infection, the encoded EGT enzyme inactivates the insect moulting hormone 20-hydroxyecdysone (20E) via the addition of a UDP-sugar group (either UDP-glucose or UDP-galactose) [15,16]. Due to the inactivation of the moulting hormone 20E, larval moulting is inhibited and infected larvae continue to eat. Finally, this may lead to a higher yield of viral progeny [17]. For several virus-host combinations, EGT has also been reported to extend the time to death of infected larvae. For example, 4th and 5th instars of *Spodoptera frugiperda* infected with wild-type (WT) AcMNPV survived longer than larvae infected with a mutant AcMNPV lacking the *egt* gene [17]. However, many other host and virus factors also have an effect from the *egt* gene on host time to death [10,18]. In another study involving *S. exigua* and *T. ni* larvae (3rd instars) the AcMNPV *egt* gene did not prolong the time to death, since larvae infected with WT AcMNPV or with an *egt-*deletion mutant did not differ significantly in the time to death [10,19].

The beet armyworm *S. exigua* is a polyphagous pest insect distributed worldwide. *S. exigua* MNPV (SeMNPV) is a specialist baculovirus that is highly infectious to its only host, *S. exigua*. [20]. A recent study by van Houte *et al.* [11] showed that SeMNPV induced tree-top disease in *S. exigua* larvae. The induced tree-top disease was light-dependent as was shown in an experimental set-up: In glass jars containing mesh wire, infected larvae moved towards light prior to death. Larvae died at elevated positions (top parts in the jar) when light was applied from above, while larvae died at low positions (bottom of the jar) when light was applied from below. The vertical position of uninfected larvae was not light-dependent, since movement patterns of uninfected larvae in light and dark conditions were similar [11].

In the current study, the role of the *egt* gene in SeMNPV-induced tree-top disease was analysed. Virus-infected larvae were placed individually in the jars and the vertical positions of these larvae were monitored twice a day until they died. We show that WT-infected *S. exigua* larvae climbed up prior to death and died at elevated positions, while larvae infected with an SeMNPV mutant lacking the *egt* gene did not climb up prior to death. Moreover, we show that this difference can be explained by an earlier death of larvae infected with the *egt-*negative virus. These larvae succumb to the virus infection prior to the onset of climbing to elevated positions in WT-infected larvae. Therefore we hypothesize that in this virus-host system, EGT facilitates tree-top disease by prolonging the time to death.

## 2. Experimental Section

### 2.1. Insect Larvae

*Spodoptera exigua* larvae were reared on artificial diet at 27 °C with 50% relative humidity as described before [21], using a 14 h light/10 h dark photoperiod (7:00 lights on, 21:00 lights off).

### 2.2. SeMNPV Virus Strains and Generation of Recombinant Bacmids

**Figure 1:**
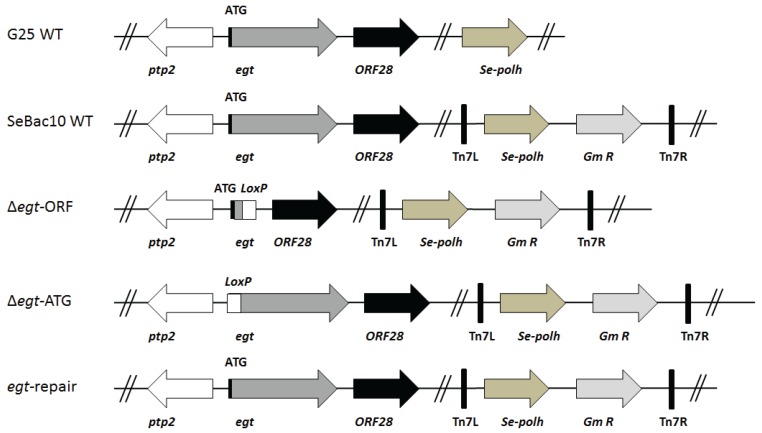
Overview of the recombinant bacmids used in this study. Two SeMNPV WT (G25 WT and SeBac10 WT) viruses were used: G25 WT is a naturally occurring strain and SeBac10 WT is a bacmid derived from the SeMNPV US1 strain. Using the SeBac10 WT bacmid, two SeMNPV mutants (Δ*egt*-ORF and Δ*egt*-ATG) were made: in Δ*egt*-ORF, a major part of the *egt* ORF was replaced by a fragment containing a mutant *loxP* site (used to create the deletion); in Δ*egt*-ATG, only the start codon was replaced by the fragment with the *loxP* site. An *egt*-repair bacmid was also constructed. For all bacmid-derived viruses (which lack the original polyhedrin gene), the *Se-polh* gene and a gentamicin resistance gene (*Gm R*) were inserted into the genomes between the left and right insertion sites, indicated as Tn7L and Tn7R, present in the bacmid. Positions of neighbouring genes (*ptp2*: protein tyrosine phosphatase 2 and ORF28) of *egt* are indicated.

In this study, two SeMNPV WT viruses were used: a naturally occurring strain (SeMNPV G25, referred to as G25 WT) [22] and viruses produced from the SeMNPV US1 strain-derived bacmid (see below; referred to as SeBac10 WT) [23]. The SeBac10 WT bacmid was used to construct two SeMNPV mutants with deletions in the *egt* gene (Figure 1). The first mutant had a deletion of the major part of the *egt* open reading frame (ORF), ranging from nucleotide 40 to 1572 (Δ*egt*-ORF), while in the second mutant only the start codon of the *egt* ORF was deleted (Δ*egt*-ATG). Mutants were created following the protocol described by Ros *et al.* [10], using primers 1 to 4 for the homologous recombination (Supplementary Table S1). The followed procedure first led to the replacement of the *eg*t ORF or start codon by a chloramphenicol resistance gene (*cat*) flanked by modified *loxP* sites. Subsequently, the *cat* gene was removed by Cre-recombinase, leaving an inserted segment of 162 bp containing the recombined *loxP* site [10,24] (Figure 1). The deletion of the *cat* gene from both constructs was checked by PCR using primers 5 and 6 (for ∆*egt*-ORF) or 7 and 8 (for Δ*egt*-ATG) (Supplementary Table S1).

After deletion of the target segments, the resulting bacmids were purified and used to transform *E. coli* DH10ΔTn7 cells carrying the transposition helper plasmid pMON7124 [25] (derived from *E. coli* DH10Bac ΔTn7 [26] by removing the original bacmid). To enable oral infection of *S. exigua* larvae, the SeMNPV polyhedrin (*polh*) promoter and ORF were re-introduced into the SeBac10 WT (which lacks the *polh* gene), Δ*egt-*ORF and Δ*egt*-ATG genomes using the Bac-to-Bac transposition protocol [25]. To this aim, a modified pFastBacDual vector (pFBD-Sepolh-Δp10) was used as the donor vector. To generate this vector, the pFastBacDual vector (Invitrogen, Bleiswijk, the Netherlands) was first modified as described in Peng *et al.* [27] by inserting the AcMNPV *polh* ORF downstream of the AcMNPV *polh* promoter and removing the *p10* promoter. Subsequently, the AcMNPV *polh* promoter and ORF were replaced by the SeMNPV *polh* promoter and ORF (corresponding to nucleotides 135474 to 776 of the SeMNPV genome [28]) using the *Xho*I and *Pst*I restriction sites in the multiple cloning site.

To ensure that a possible phenotype of the Δ*egt* viruses was not due to any other genome mutations, a repair virus (*egt*-repair) was constructed, using the Δ*egt*-ORF bacmid as a backbone (Figure 1). For the construction, a fragment corresponding to nucleotide 26091 to 28544 of the SeMNPV genome and covering the entire *egt* ORF was amplified with Phusion polymerase (Finnzymes, Fisher Scientific, Landsmeer, the Netherlands) using primers 9 and 10 (Supplementary Table S1). Purified PCR products (100 ng/µL) were mixed with Δ*egt*-ORF bacmid DNA (500 ng/µL) and Lipofectin transfection reagent (Invitrogen) in a 1:1:1 volume ratio and incubated at room temperature for 15 min. The mixture was used to inject 4th instars of *S. exigua* to facilitate generation of the *egt* gene repair virus via homologous recombination. To this end, each larva was injected with 10 µL of the mixture using a Humapen Luxura insulin injection pen (Lilly, Houten, the Netherlands) and placed in a 12-well plate with a piece of artificial diet. The larvae were incubated at 27 °C until the larvae had liquefied. Viral occlusion bodies (OBs) were purified from the cadavers and viral DNA was extracted as described in Simon *et al.* [29]. Extracted viral DNA was used to transform *E. coli* cells and primers 5 and 6 (Supplementary Table S1) were used to screen the *E. coli* colonies for the presence of the correct repair bacmid via PCR.

### 2.3. Generation, Amplification and Purification of Viruses

Bacmid DNA was isolated from *E. coli* DH10ΔTn7 cells carrying the various bacmids (SeBac10 WT, Δ*egt*-ORF, Δ*egt-*ATG or *egt*-repair) using the PureLinK HiPure Plasmid Midiprep Kit (Invitrogen). Bacmid DNA (500 ng/μL) and Lipofectin transfection reagent (Invitrogen) were mixed in a 2:1 volume ratio. The mixture was incubated at room temperature for 15 min and used to inject 4th instars as described above. After injection, larvae were placed in a 12-well plate with a piece of artificial diet and incubated at 27 °C until the larvae liquefied. Liquefied larvae were ground and mixed with 10% sucrose solution containing 0.4% (w/v) Patent Blue V Sodium salt colouring dye (Sigma-Aldrich, Zwijndrecht, the Netherlands). To amplify the obtained viruses, the sucrose-virus suspension was used to orally infect 3rd and 4th instars of *S. exigua* using droplet feeding as described before [6]. After liquefaction, larvae were ground in water and filtered through a double layer of cheese cloth. The suspension was centrifuged at 500× *g* for 5 min to remove larval debris, after which the supernatant was centrifuged at 4000× *g* for 30 min to pellet the viral occlusion bodies (OBs). Finally, the OBs were resuspended in water and stored at 4 °C. The concentration of OBs was counted using a Bürker-Türk haemocytometer (Marienfeld, Lauda-Königshofen, Germany).

### 2.4. Infectivity Assays

Infectivity assays were performed to determine the infectivity for each virus, as described in Ros *et al.* [10]. Late 2nd instars of *S. exigua* were starved overnight for 16 h and allowed to moult during starvation. Newly moulted 3rd instars were selected and infected using droplet feeding as described in van Houte *et al.* [6]. Five viruses (G25 WT, SeBac10 WT, ∆*egt*-ORF, ∆*egt*-ATG, *egt*-repair) were used to infect larvae and five different concentrations (6-fold dilutions: 1.0 × 10^3^, 6.0 × 10^3^, 3.6 × 10^4^, 2.0 × 10^5^ and 1.3 × 10^6^ OBs/mL) were used for each virus (the dilutions were freshly prepared from a virus stock for each virus on the day of infection). For each virus concentration, 24–36 larvae were infected. Mock-infected larvae, which were droplet fed with a virus-free sucrose solution, were used as controls. Starting from two days post infection (dpi), larvae were scored for mortality until all larvae had died or had pupated. The assays were performed three times as three independent replicates. Data were analysed using a logistic regression analysis in the program R v3.0.0 [10]. Treatment was used as a fixed effect and the model followed a binomial distribution. In addition, the 50% lethal concentration (LC_50_) values were obtained using a logistic regression analysis in the program PoloPlus v1.0 (LeOra Software, 2002) [30].

### 2.5. Mortality Assays

Mortality assays were performed to determine the effect of each virus (G25 WT, SeBac10 WT, ∆*egt*-ORF, ∆*egt*-ATG, *egt*-repair) on the time to death of the infected larvae. 3rd instars of *S. exigua* were infected with a viral concentration of 10^6^ OBs/mL (known to kill at least 90% of infected larvae, the same concentration as used in the behavioural assays below), using droplet feeding as described above [6]. Mock-infected larvae were used as controls. Infected larvae were checked twice per day until they had died or had pupated (mock). For each virus, 36 larvae were infected and assays were performed three times as three independent replicates. The effects of the factors (treatment and experiment) on time to death were analysed using Cox’s proportional hazards model in the program R, as described in Ros *et al.* [10]. Since almost all larvae died as 3rd instars (or while moulting from 3rd to 4th instar), larval stage was not included as a factor in the model. In addition, mean time-to-death (MTD) values were calculated in R.

### 2.6. Behavioural Assays to Measure Tree-Top Disease

Two behavioural assays, each with a different set of viruses, were done and each assay was performed twice as two independent replicates. In the first assay, larvae infected with the viruses G25 WT, SeBac10 WT, ∆*egt-*ORF or *egt*-repair were compared to investigate whether the *egt* gene plays a role in tree-top disease in *S. exigua* larvae. In the second assay, larvae infected with the viruses SeBac10 WT, ∆*egt*-ORF or ∆*egt-*ATG were compared to determine whether the EGT protein is required for tree-top disease. For all assays, newly moulted 3rd instars of *S. exigua* were infected with either a virus or a mock solution (containing no virus), using droplet feeding as described before [6]. For all viruses, a concentration of 10^6^ OBs/mL was used, a concentration known to kill at least 90% of infected larvae. For each assay, 30–40 larvae were infected (10 for the mock). Infected larvae were placed individually in glass jars (120 mm high and 71 mm in diameter) which were covered with a piece of transparent plastic Saran wrap (Supplementary Figure S4). Sterile mesh wire was placed in the jars to facilitate climbing and a piece of artificial food was placed at the bottom of the jars. Jar walls were protected from light using aluminum foil. Light was provided from above using three luminescent tube lamps of 18 Watts each placed at a 30 cm distance above the jars. Jars were placed at 27 °C with 50% relative humidity and a 14:10 LD photoperiod (7:00 lights on, 21:00 lights off). The vertical position of the larvae (highest point of the larval body) in the jars was monitored twice a day (mainly at 8:00–10:00 and 20:00–22:00, for the exact time points see graphs) from one dpi until all larvae had died or pupated. Larvae that did not die due to viral infection were excluded from the data analyses.

The position at death was analyzed using a linear regression model (lm) analysis in the program R [10]. Treatment (G25 WT, SeBac10 WT, Δ*egt*-ORF, Δ*egt*-AGT and *egt*-repair) and experiment (two replicates) were used as explanatory factors and it was determined whether these factors affected the positions at death. Since almost all larvae died as 3rd instar (or while moulting from 3rd to 4th instar), larval stage was not included as a factor.

### 2.7. RNA Extraction and RT-PCR

Newly moulted 3rd instars *S. exigua* were infected by droplet feeding with a viral concentration of 10^6^ OBs/mL (G25 WT, SeBac10 WT, ∆*egt-*ORF, ∆*egt-*ATG and *egt*-repair virus) as described before [6]. Mock-infected larvae were used as controls. At two dpi single larvae were homogenized in 250 µL Trizol reagent (Invitrogen) and total RNA was isolated following the manufacturer’s instructions. The RNA pellet was dissolved in 50 µL water and heated for 10 min at 55 °C. Any contaminating DNA was removed with the DNAfree kit (Applied Biosystems, Life Technologies, Bleiswijk, the Netherlands). cDNA was produced using SuperScript III Reverse Transcriptase (Invitrogen) following the company’s protocol. RT-PCR was performed, using primer pairs to amplify (A) a 468 bp sequence within the SeMNPV *egt* gene to check for the deletion of part of the *egt* ORF (primers 15 and 16, Supplementary Table S1), (B) a 899 bp sequence containing the *egt* start codon to check for the correct deletion of the start codon (forward primer annealed 6 to 27 bp upstream of the *egt* start codon, reverse primer annealed within the *egt* ORF) (primers 9 and 16, Supplementary Table S1), (C) a 492 bp sequence within the SeMNPV *ie1* ORF to check for a successful virus infection (primers 13 and 14, Supplementary Table S1), and (D) a 486 bp sequence within the *S. exigua* host translation initiation factor *eIF5A* ORF [31] to check for successful RNA extraction and cDNA production for all larvae (primers 11 and 12, Supplementary Table S1). For each sample, a control sample was run in which the RT step was omitted (non-RT) to check for any DNA contamination. In addition, for each PCR a negative control with only water as template was processed.

## 3. Results

### 3.1. Viral Infectivity of the Generated Mutants

To study whether deletion of the viral *egt* gene or of the *egt* start codon from the SeMNPV genome affected viral infectivity, we performed a logistic regression on the mortality data obtained after infection of *S. exigua* 3rd instars with G25 WT, SeBac10 WT, ∆*egt*-ORF, ∆*egt*-ATG or *egt*-repair viruses, for each replicate separately. For each virus, an odds ratio (relative potency) was determined: the ratio of infectivity of the respective virus compared to the G25 WT virus. In the first replicate, the infectivity of the WT virus differed from the infectivity of the mutant viruses (∆*egt*-ORF, ∆*egt*-ATG) and the *egt*-repair virus (judged by a lack of overlap of 95% confidence interval of the odds ratio and *p* < 0.001; Supplementary Table S2A), with the mutant and repair viruses being more virulent than the WT virus (Supplementary Table S2A). However, this difference was absent in the other two replicates (odds ratios were not significantly different; Supplementary Table S2A). In the second replicate, the repair was slightly more virulent than the G25 WT virus (Supplementary Table S2A), but this effect was absent in the other two replicates. The LC_50_ value for each of the viruses is given in Supplementary Table S2B. We chose to use a high virus concentration (killing at least 90% of all larvae) for infections in the behavioural assays, which came down to a concentration of 10^6^ OBs/mL for each virus.

### 3.2. The SeMNPV egt Gene Extends the Time to Death of S. exigua Larvae

We investigated whether removal of the viral *egt* gene or of the *egt* start codon affected the time to death for 3rd instars of *S. exigua*, using a survival analysis. The model used (Cox’s proportional hazards model) determines a mortality rate (hazard rate, rate at which larvae died) for the different values of the factors (treatment and experiment). The mortality rate was significantly affected by the treatment. The mortality rate for larvae infected with the mutant viruses was 2.04 (∆*egt*-ORF, z = 5.00, *p* < 0.001) and 1.40 (∆*egt*-ATG, z = 2.41; *p* = 0.016) times higher than for G25 WT-infected larvae. Similar ratios were found when compared to SeBac10 WT (1.90 for ∆*egt*-ORF, z = 4.52, *p* < 0.001 and 1.31 for ∆*egt*-ATG, z = 1.95, *p* = 0.052) or to *egt*-repair (2.37 for ∆*egt*-ORF, z = 5.97, *p* < 0.001 and 1.63 for ∆*egt*-ATG, z = 3.46, *p* < 0.001). This is reflected in higher mean time to death (MTD) values for the G25 WT (73.84 h), SeBac10 WT (73.51 h) and *egt*-repair (76.48 h) viruses compared with the ∆*egt*-ORF (64.56 h) and ∆*egt*-ATG (68.95 h) viruses (Supplementary Table S2B). There was no significant difference between the three replicates.

### 3.3. Deletion of the egt Gene Prevents SeMNPV-Induced Tree-Top Disease

**Figure 2:**
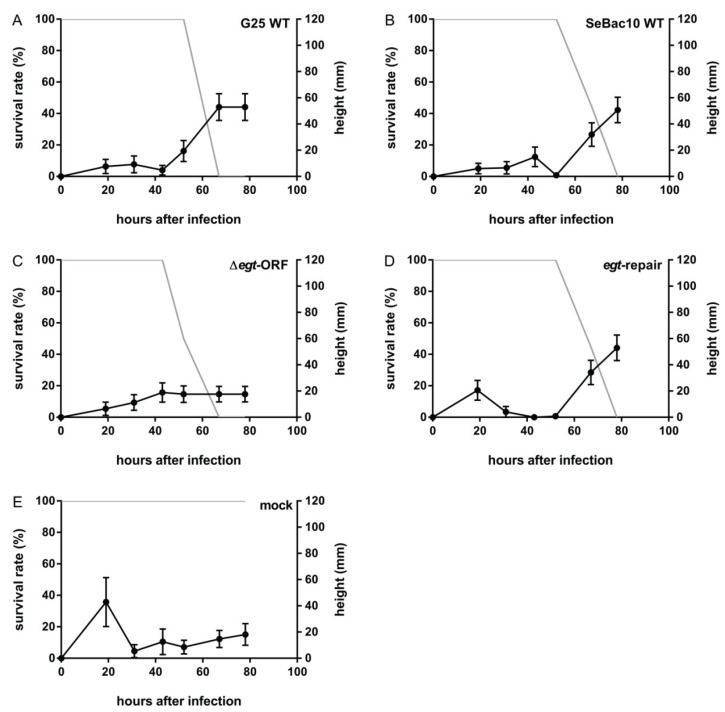
The effect of the deletion of the *egt* ORF on SeMNPV-induced tree-top disease in *S. exigua* larvae. Percentage surviving larvae (grey line) and height (mm) of larvae or cadavers (black line) were recorded at different time points after infection for 3rd instar *S. exigua* larvae infected with G25 WT (**A**, *n* = 25), SeBac10 WT (**B**, *n* = 27), Δ*egt-*ORF (**C**, *n* = 24), *egt*-repair (**D**, *n* = 29) or mock (**E**, *n* = 10). Error bars represent the standard error of the mean (SEM).

To investigate the role of the SeMNPV *egt* gene in SeMNPV-induced tree-top disease, a behavioural assay was performed using 3rd instars of *S. exigua* infected with G25 WT, SeBac10 WT, Δ*egt*-ORF or *egt*-repair viruses. Both SeMNPV WT strains (G25 WT and SeBac10 WT) induced tree-top disease in *S. exigua* larvae. Infected larvae stayed at low positions until 52 h post infection (hpi) and from this time point on, larvae climbed up and finally died at elevated positions (Figure 2A,B and Supplementary Figure S2A,2B). When the *egt* ORF was deleted, infected larvae died at low positions (Figure 2C and Supplementary Figure S2C, black lines) (Δ*egt*-ORF (*n* = 52) *versus* G25 WT (*n* =54) and SeBac10 WT (*n* = 56); *T*-test = 3.859 and 4.815, respectively; d.f. = 216; *p* < 0.001 for both comparisons). The Δ*egt*-ORF mutant-infected larvae died earlier (from 43 hpi) than both WT-infected larvae (from 67 hpi), and in fact almost all were dead before the time point (52 hpi) at which WT-infected larvae started their upwards movement. Reintroducing the *egt* gene into the Δ*egt*-ORF genome (*egt*-repair) restored the WT phenotype, with *egt*-repair-infected larvae dying at similar positions (*egt*-repair (*n* = 59) *versus* G25 WT and SeBac10 WT; *T*-test = −0.168 and 0.764, respectively; d.f. = 216; *p* = 0.853 and 0.446, respectively) and at a similar time point as the WT-infected larvae (from 67 hpi onwards) (Figure 2D and Supplementary Figure S2D, grey lines). Mock-infected larvae stayed at low positions throughout the experiment (Figure 2E and Supplementary Figure S2E). No significant differences were found between the two replicate experiments (*T*-test = −0.012; d.f. = 216; *p* = 0.990).

### 3.4. The EGT Protein but not the egt Transcript Is Involved in This Process

Deletion of the *egt* ORF may only affect EGT protein synthesis, but it may additionally affect neighbouring gene functioning. Moreover, a precursor miRNA encoding sequence has been predicted within the *egt* ORF from position nt 28046 to 28136 (database: Vir-Mir) [32]. A viral miRNA could play a role in the pathology of the virus or may regulate host physiology. We therefore created a mutant virus lacking only the *egt* start codon, but still containing the rest of the ORF, including the predicted precursor miRNA sequence. This allowed us to investigate whether the complete ORF deletion is needed for the observed differences in larval behaviour or whether this is also achieved when only the start codon is absent. In the Δ*egt*-ATG mutant, the ATG start codon was replaced by an insert of 162 bp including the mutant *loxP* site (see Materials and Methods). This insert still allowed the *egt* transcript to be formed (as confirmed in Figure 4B), while translation of the EGT protein was inhibited. The next in-frame ATG codon is 223 bp downstream of the deleted ATG. In the unlikely event that the next in-frame ATG would be used, 75 amino acids (aa) from the N-terminal end of the EGT protein would be removed (the *egt* gene is 524 aa in size, removal of 75 aa would be a considerable truncation of the functional protein). If the behavioural changes are observed for both mutants (Δ*egt*-ORF and Δ*egt*-ATG), it is likely that the encoded EGT protein is involved. If changes are only observed for larvae infected with the Δ*egt*-ORF mutant, but not for larvae infected with the Δ*egt*-ATG mutant, there might be a role for the transcript, the encoded miRNA or even for side-effects on neighbouring genes.

**Figure 3:**
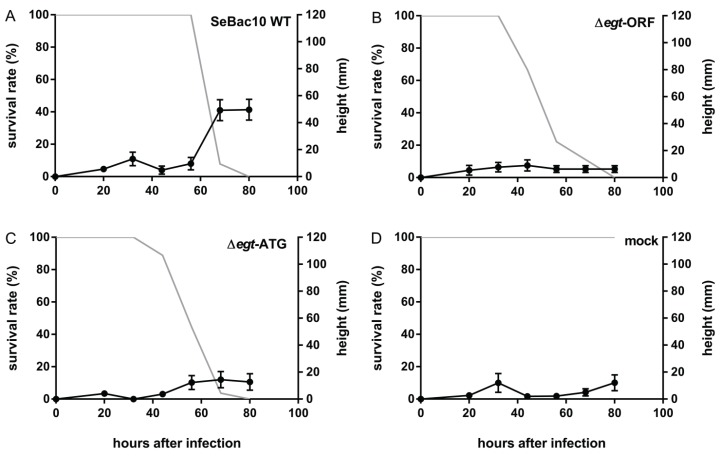
The effect of the deletion of the *egt* start codon on SeMNPV-induced tree-top disease in *S. exigua* larvae. Percentage surviving larvae (grey line) and height (mm) of larvae or cadavers (black line) were recorded at different time points after infection for 3rd instar *S. exigua* larvae infected with SeBac10 WT (**A**, *n* = 38), Δ*egt-*ORF (**B**, *n* = 27), Δ*egt-*ATG (**C**, *n* = 27) or mock (**D**, *n* = 10). Error bars represent the standard error of the mean (SEM).

Larvae infected with SeBac10 WT, Δ*egt*-ORF and Δ*egt*-ATG were compared. SeBac10 WT-infected larvae stayed at low positions until 56 hpi (Figure 3A and Supplementary Figure S3A). They displayed climbing behaviour prior to death and finally died at elevated positions. Larvae infected with Δ*egt*-ORF or Δ*egt*-ATG virus (Figure 3B,3C and Supplementary Figure S3B,S3C, black lines) did not climb up prior to death and died at low positions (SeBac10 WT (*n* = 66) *versus* Δ*egt*-ORF (*n* = 53) and Δ*egt*-ATG (*n* = 57); *T*-test = −7.109 and −7.020, respectively; d.f. = 172; *p* < 0.001 for both comparisons). These mutant-infected larvae died earlier (from 32 hpi onwards) than WT-infected larvae (from 56 hpi onwards). Because Δ*egt*-ORF and Δ*egt*-ATG virus-infected larvae behaved similarly, we conclude that the EGT protein is required for tree-top disease.

### 3.5. The Egt Gene Is Expressed in WT and Repair Viruses

**Figure 4:**
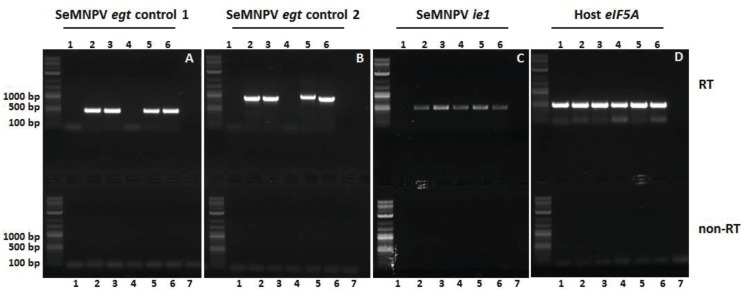
RT-PCR analysis of *Spodoptera exigua* larvae infected with WT, mutant and repair viruses. Larvae were mock-infected (1) or infected with G25 WT (2), SeBac10 WT (3), Δ*egt*-ORF (4), Δ*egt*-ATG (5) or *egt*-repair (6) viruses and processed for RT-PCR analysis at two dpi. For each PCR a water control (7) was included. The presence or absence of SeMNPV *egt* gene transcripts was checked by RT-PCR (upper panels), using two different primer pairs, one pair annealing within the *egt* ORF (**A**) and the second pair annealing to the 5' upstream region of *egt* and within the *egt* ORF (**B**). Expression of the SeMNPV *ie1* gene (**C**) was used as infection control and expression of the S. *exigua eIF5A* gene (**D**) was used as cDNA quality control. For each RT sample, a duplo sample without RT step (non-RT) was performed in parallel (lower panels). The 2-Log DNA Ladder (0.1–10.0 kb, New England BioLabs Inc., Leiden, the Netherlands) was used in the agarose gel to estimate PCR fragment sizes.

To confirm the expression of *egt* in WT- (G25 WT, SeBac10 WT), repair- (*egt*-repair) and Δ*egt-*ATG-infected larvae, and to confirm the absence of *egt* expression in ∆*egt*-ORF-infected larvae, an RT-PCR amplification was performed on total RNA extracted from mock- and virus-infected single whole larvae at two dpi (Figure 1 and Figure 4). As expected, *egt-*specific mRNA was detected using primers annealing within the *egt* ORF (primers 15 and 16, Supplementary Table S1) in larvae infected with G25 WT, SeBac10 WT, ∆*egt*-ATG and *egt* repair viruses (Figure 4A; lanes 2–3 and 5–6), but was absent in mock- and ∆*egt*-ORF-infected larvae (Figure 4A, lanes 1 and 4). With primers annealing to the 5' upstream region of the *egt* ORF and to the 3' end of the *egt* ORF (primers 9 and 16, Supplementary Table S1) it was shown that the ATG start codon of the *egt* gene was replaced by an insert of 162 bp including the mutant *loxP* site in the ∆*egt*-ATG mutant, resulting in a slightly larger product (Figure 4B, lane 5) than for the WT and repair constructs (lanes 2–3 and 6). As expected, no product was seen for mock- and ∆*egt*-ORF-infected larvae (Figure 4B, lanes 1 and 4). An RT-PCR on the SeMNPV *ie1* transcript was used as a control for virus infection and showed that the *ie1* gene was indeed expressed in all virus-infected individuals, but not in the mock-infected larvae (Figure 4C). The host *S. exigua eIF5A* gene was expressed in all mock- and virus-infected larvae (Figure 4D), confirming successful RNA isolation and cDNA production.

## 4. Discussion

Manipulation of host behaviour is a common strategy among parasites to enhance their survival and/or transmission [3,4,5]. The underlying mechanisms of various examples of parasitic manipulation are now starting to be unravelled. It has been reported for more than a century that baculoviruses can induce tree-top disease in their lepidopteran hosts [3,8], but it was only in 2011 that the *egt* gene from LdMNPV was reported to be involved in this process in *L. dispar* larvae [9]. A recent study showed that the specialist baculovirus SeMNPV induces light-dependent tree-top disease in *S. exigua* larvae [11]. The role of the SeMNPV *egt* gene in this process was, however, still unknown. In this study, we showed that the SeMNPV *egt* gene is required for the induction of tree-top disease in 3rd instars of *S. exigua*. Furthermore, we observed in our studies that the absence of tree-top disease in larvae infected with the *egt-*mutant viruses is a consequence of the fact that these larvae died earlier than WT-infected larvae, prior to the onset of the pre-death climbing behaviour.

Our study reveals that in SeMNPV-infected *S. exigua* larvae, EGT facilitates tree-top disease via prolonging the larval time to death. SeMNPV EGT extended the time to death of infected larvae, and in that extended life time the WT-infected caterpillars moved upwards, while the larvae infected with *egt*-mutants had already succumbed to virus infection. Previously, we showed that SeMNPV-induced tree-top disease is light-dependent and suggested that the virus might induce climbing by making use of host pathways involved in phototaxis and/or light perception [11]. Whether SeMNPV EGT plays a further role in the induction of the light-dependent tree-top disease in *S. exigua* larvae remains undetermined. EGT has been reported to extend the host’s life span in several virus-host systems, although in some systems no effect, or even a reduced time to death has been found [10,13,18]. Apparently, a variety of factors, including the virus-host interaction studied, the timing of infection, the developmental stage of the larvae and the viral concentration used, may have effects on the influence of the *egt* gene on host time to death [10,18].

Strikingly, in AcMNPV-infected *S. exigua* larvae, tree-top disease appeared to be moulting-dependent: only larvae that moulted during the course of infection climbed up and died at elevated positions [10,33]. Those larvae that did not undergo moulting died at low positions. This observation appears to be specifically induced by AcMNPV in *S. exigua* larvae, since it was not observed in the current study with SeMNPV-infected *S. exigua* larvae and also not with AcMNPV in *T.ni* larvae [10]. Here, most larvae died as 3rd instar or while moulting from 3rd to 4th instar and died at elevated positions.

We performed an additional experiment including a mutant SeMNPV virus lacking only the *egt* start codon (Δ*egt*-ATG). This experiment showed, combined with the observation that both the *egt* ORF and the *egt* start codon mutant reduced the time to death and prevented tree-top disease in infected larvae, that the EGT protein is most likely responsible for extending the time to death and allowing pre-death climbing behaviour. It has been shown that behavioural changes in insects can be influenced by miRNA pathways or miRNA levels [34] and it has been reported that miRNAs might be involved in regulating behaviour in *Drosophila* [35]. Similarly, viruses might affect host behaviour using viral miRNAs. A precursor miRNA encoding sequence has been predicted within the *egt* ORF [32]. However, our data indicate that the *egt* transcript is not involved in the altered phenotype. The RT-PCR results demonstrated that the *egt* transcript was still formed and the region for which a miRNA was predicted was still intact in this mutant. Whether the miRNA encoded in the *egt* gene is produced and whether it has a regulating role in the infection process must still be investigated.

The *egt* gene is present in almost all lepidopteran baculoviruses except in one clade of granuloviruses [10]. Several studies have now investigated the role of *egt* in baculovirus-induced tree-top disease. Although the effects of *egt* on the occurrence of tree-top disease have been observed (Hoover *et al.* [9] and this study), it is absent in other systems (Ros *et al.* [10]) and its exact role remains unclear. Possibly, *egt* exerts its effect on tree-top disease by affecting the time to death and/or by inhibiting moulting-related climbing behaviour [10]. In the current study, *egt* extends the time to death, and *egt*-mutant-infected larvae do not reach the time point at which in WT-infected larvae tree-top disease is observed. Apart from affecting the time to death of larvae, *egt* is also known to suppress host moulting [15,16]. Larval moulting is often accompanied by changes in behaviour, and by affecting moulting, *egt* might also change moulting-related behaviour. An example was seen in AcMNPV-infected 3rd instars of *T. ni* and *S. exigua*, where *egt* did affect moulting-related climbing behaviour but not tree-top disease, which occurred much later than the moulting-related climbing [10]. If the death of larvae occurred around the time that larvae moult, or are about to moult, *egt* might, by having an inhibitory effect on moulting, also affect moulting-related behaviour, which can give the appearance that *egt* induces tree-top disease. This might be the case in LdMNPV-infected *L. dispar* larvae [9,10]. *Lymantria dispar* larvae feed in the tree canopy and move downwards to moult. In LdMNPV WT-infected larvae moulting was suppressed and larvae died up in the tree, while larvae infected with the LdMNPV Δ*egt* virus moved downwards to moult and died at low positions. In the case of LdMNPV, the apparent effect of *egt* on tree-top disease might be a consequence of its inhibitory effect on moulting and on moulting-related behaviour.

## 5. Conclusions

In this study we examined the role of the *egt* gene in SeMNPV-induced tree-top disease. We found that larvae infected with a mutant virus lacking the *egt* gene presented a shorter time to death and died before the onset of pre-death climbing behaviour. Moreover, deletion of either the open reading frame or the ATG start codon of the *egt* gene prevented tree-top disease, indicating that the EGT protein is required for the occurrence of tree-top disease. We observed that EGT facilitates the pre-death climbing behaviour by prolonging the larval time to death. Most likely, additional viral genes are needed to induce tree-top disease.

## Supplementary Files

Supplementary File 1
