# Supplementary material for: Parasitic Manipulation of Host Behaviour: Baculovirus SeMNPV EGT Facilitates Tree-Top Disease in Spodoptera exigua Larvae by Extending the Time to Death
Source: Insects. 2015 Jul 31;6(3):716–31. doi: 10.3390/insects6030716 (PMC4598661; doi:10.3390/insects6030716)
Supplement: Supplementary File 1 [file insects-06-00716-s001.pdf]

Supplementary Materials

Table S1. Primers used in this study.

Number	Primer Name	Sequence (5'-3')	Function
1	Δ*egt*-ORF-Fw	*CATCGGTCACCATGAACGGTTGCGCTGTCCTAATTTTATTTTTTGCACTG*GCTCGGATCCACTAGTAACGG	Primer with 50 bp overhang (italics) to amplify a product for homologous recombination to remove part of the *egt* ORF
2	Δ*egt*-ORF-Rv	*ATTTAGGGTTAAATTACATGGTTCATACATACACACACATCCTGTTTTTT*CCTCTAGATGCATGCTCGAG	Primer with 50 bp overhang (italics) to amplify a product for homologous recombination to remove part of the *egt* ORF
3	Δ*egt*-ATG-Fw	*TTTAGGTCACCCATTTACTGTATCGAATCATCGGTCACCGCTCGGATCCA*CTAGTAACG	Primer with 50 bp overhang (italics) to amplify a product for homologous recombination to remove the *egt* start codon
4	Δ*egt*-ATG-Rv	*GCCGAACCCGTGGTCAGTGCAAAAAATAAAATTAGGACAGCGCAACCGTT*CCTCTAGATGCATGCTCG	Primer with 50 bp overhang (italics) to amplify a product for homologous recombination to remove the *egt* start codon
5	Δ*egt*-ORF-check Fw	ATGTGTGCTCTTCGTCAGATG	To check the Δ*egt*-ORF deletion mutant
6	Δ*egt*-ORF-check Rv	TATTGCCTACGCGCGC	To check the Δ*egt*-ORF deletion mutant
7	Δ*egt*-ATG-check Fw	GGCTAAACCGATGTTGTAGTG	To check the Δ*egt*-ATG deletion mutant
8	Δ*egt*-ATG-check Rv	CCCGGTACCTCACACTAAATTAATTCTCAGTAATTGAC	To check the Δ*egt*-ATG deletion mutant
9	*egt*-repair-Fw	ATTTACTGTATCGAATCATCGG	To amplify a product for homologous recombination to generate the *egt*-repair virus, for RT-PCR on SeMNPV *egt* and to check *egt*-ATG deletion
10	*egt*-repair-Rv	GGGTTAAATTACATGGTTCATACA	To amplify a product for homologous recombination to generate the *egt*-repair virus
11	Se-*eIF5A*-Fw	GCCATGGCTGACATCGAGGATAC	RT-PCR on *S. exigua eIF5A*
12	Se-*eIF5A*-Rv	GCGGTACCGGTTTATTTGTCGAGAGC	RT-PCR on *S. exigua eIF5A*
13	Se-*ie1* Fw	GACAAGAATGACGATGATATCGG	RT-PCR on SeMNPV *ie1*
14	Se-*ie1* Rv	GGACAATTGCTTTTCCGAAAAC	RT-PCR on SeMNPV *ie1*
15	Se-*egt*-ORF-Fw	CAAGAGGTTGATTGACGAACAA	RT-PCR on SeMNPV *egt* and to check *egt*-ORF deletion
16	Se-*egt*-ORF-Rv	AGCGATTTGGGATGTTTGTC	RT-PCR on SeMNPV *egt* and to check *egt*-ORF and *egt*-ATG deletion

Table S2. (A) Outcome of the logistic regression analysis of the infectivity assays. Per replicate, an odds ratio (relative potency) was determined for each virus: the ratio of the infectivity of the respective virus relative to the infectivity of the G25 WT virus. The upper and lower limits of the 95% confidence interval are also given, as well as the *P* value.

Replicate	Viruses	Odds Ratio	95% Confidence Interval		*P value*
			Low	High	
1	G25 WT	1.000	-	-	-
	SeBac10 WT	1.634	0.988	2.715	0.057
	Δegt-ORF	2.654	1.601	4.440	<0.001 ***
	Δ*egt*-ATG	5.989	3.542	10.292	<0.001 ***
	egt-repair	3.845	2.300	6.509	<0.001 ***
2	G25 WT	1.000	-	-	-
	SeBac10 WT	1.291	0.774	2.158	0.329
	Δegt-ORF	0.680	0.407	1.132	0.139
	Δ*egt*-ATG	1.135	0.679	1.902	0.629
	egt-repair	1.759	1.046	2.974	0.034 *
3	G25 WT	1.000	-	-	-
	SeBac10 WT	1.330	0.786	2.256	0.288
	Δegt-ORF	0.597	0.349	1.016	0.058
	Δ*egt*-ATG	1.607	0.946	2.745	0.080
	egt-repair	0.899	0.531	1.521	0.691

Table S2. (B) Dose-mortality response (log LC_{50}) and mean time to death (MTD) of 3rd instar *S. exigua* larvae infected with G25 WT, SeBac10 WT, Δ*egt*-ORF, Δ*egt*-ATG or *egt*-repair virus. MTD was determined for a virus concentration of 10^6 OBs/mL (approximately 90%–95% mortality).

Virus	Log LC_{50} (OBs/mL)	Log 95% Confidence Interval (OBs/mL)		MTD (h)	95% Confidence Interval (h)	
		low	high		low	high
G25 WT	4.70	4.44	4.97	73.84	71.87	75.81
SeBac10 WT	4.42	4.16	4.67	73.51	71.75	75.28
Δ*egt*-ORF	4.64	4.40	4.88	64.56	62.43	66.68
Δ*egt*-ATG	4.08	3.84	4.30	68.95	66.74	71.16
egt-repair	4.26	3.98	4.53	76.48	74.66	78.29

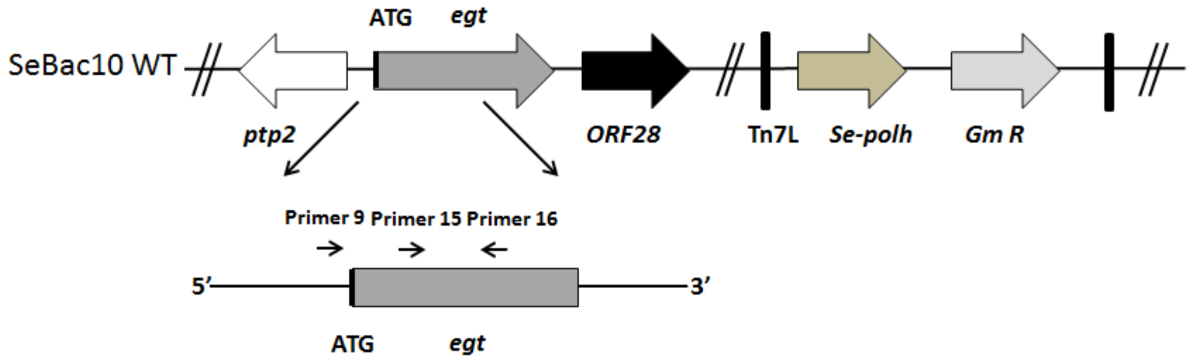

Figure S1. Position of the primers (horizontal arrows) that were used to check the deletion of the *egt* ORF or ATG start codon. Primers 15 and 16 anneal within the *egt* ORF and were used to check the deletion of the *egt* ORF. Primer 9 (anneals 27 bp upstream of the *egt* start codon) and primer 16 (anneals within the *egt* ORF) were used to check the deletion of the *egt* start codon.

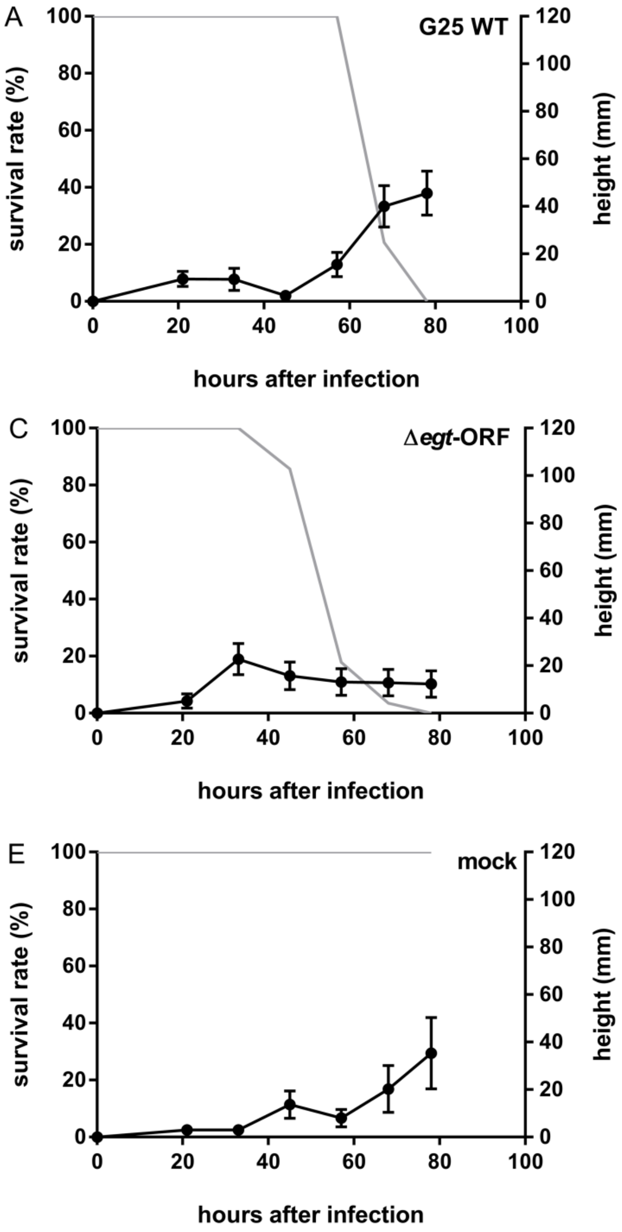

Figure S2. Repetition of the experiment presented in Figure 2. The effect of the deletion of the *egt* ORF on SeMNPV-induced tree-top disease in *S. exigua* larvae. Percentage surviving larvae (grey line) and height (mm) of larvae or cadavers (black line) were recorded at different time points after infection for 3rd instar *S. exigua* larvae infected with G25 WT (**A**, $n = 29$), SeBac10 WT (**B**, $n = 29$), Δ*egt*-ORF (**C**, $n = 28$), *egt*-repair (**D**, $n = 30$) or mock (**E**, $n = 10$). Error bars represent the standard error of the mean (SEM).

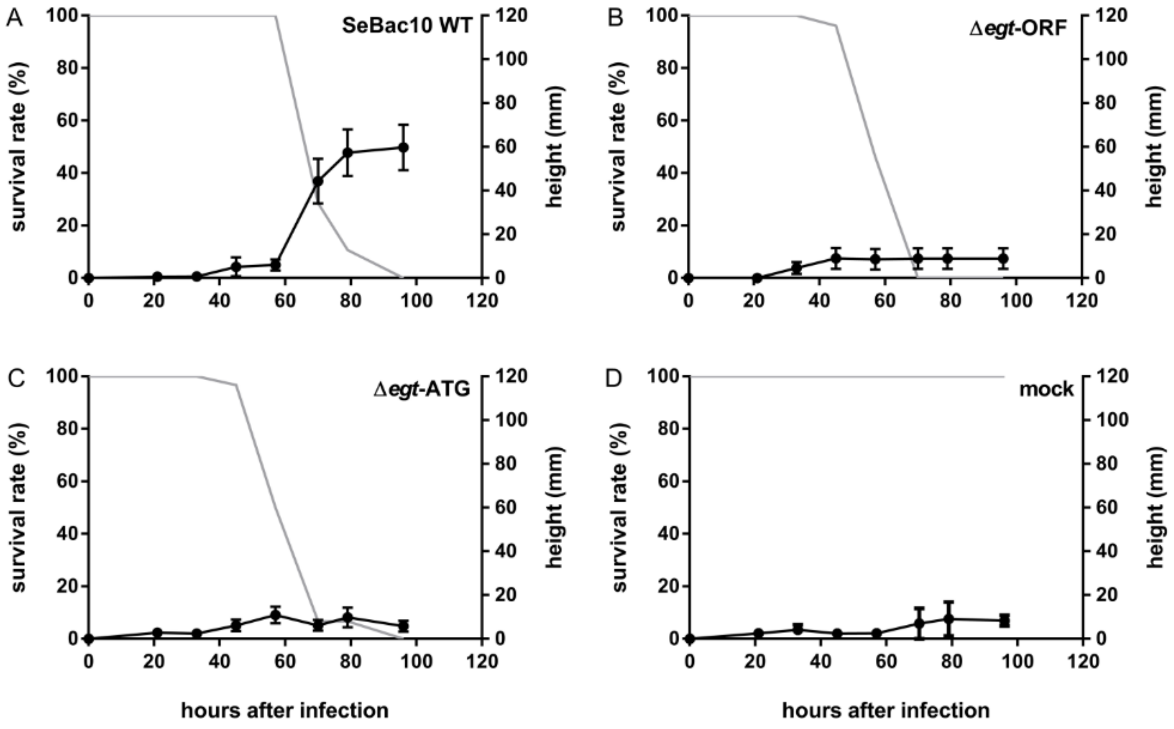

Figure S3. Repetition of the experiment presented in Figure 3. The effect of the deletion of the *egt* start codon on SeMNPV-induced tree-top disease in *S. exigua* larvae. Percentage surviving larvae (grey line) and height (mm) of larvae or cadavers (black line) were recorded at different time points after infection for 3rd instar *S. exigua* larvae infected with SeBac10 WT (**A**, *n* = 28), Δ*egt*-ORF (**B**, *n* = 26), Δ*egt*-ATG (**C**, *n* = 30) or mock (**D**, *n* = 10). Error bars represent the standard error of the mean (SEM).

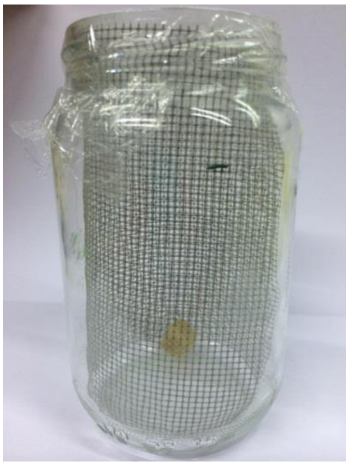

Figure S4. Experimental set-up for measuring pre-death climbing behaviour. Larvae were placed individually in glass jars (120 mm high and 71 mm in diameter). Sterile mesh wire was placed in the jars to facilitate climbing and a piece of artificial food was placed at the bottom of the jar [11].

© 2015 by the authors; licensee MDPI, Basel, Switzerland. This article is an open access article distributed under the terms and conditions of the Creative Commons Attribution license (http://creativecommons.org/licenses/by/4.0/).
